# Augmented Recognition of Distracted Driving State Based on Electrophysiological Analysis of Brain Network

**DOI:** 10.34133/cbsystems.0130

**Published:** 2024-07-04

**Authors:** Geqi Qi, Rui Liu, Wei Guan, Ailing Huang

**Affiliations:** ^1^ Key Laboratory of Transport Industry of Big Data Application Technologies for Comprehensive Transport, Ministry of Transport, Beijing Jiaotong University, Beijing, China.; ^2^ Key Laboratory of Brain-Machine Intelligence for Information Behavior—Ministry of Education, Shanghai International Studies University, Shanghai, China.; ^3^ School of Systems Science, Beijing Jiaotong University, Beijing, China.

## Abstract

In this study, we propose an electrophysiological analysis-based brain network method for the augmented recognition of different types of distractions during driving. Driver distractions, such as cognitive processing and visual disruptions during driving, lead to distinct alterations in the electroencephalogram (EEG) signals and the extracted brain networks. We designed and conducted a simulated experiment comprising 4 distracted driving subtasks. Three connectivity indices, including both linear and nonlinear synchronization measures, were chosen to construct the brain network. By computing connectivity strengths and topological features, we explored the potential relationship between brain network configurations and states of driver distraction. Statistical analysis of network features indicates substantial differences between normal and distracted states, suggesting a reconfiguration of the brain network under distracted conditions. Different brain network features and their combinations are fed into varied machine learning classifiers to recognize the distracted driving states. The results indicate that XGBoost demonstrates superior adaptability, outperforming other classifiers across all selected network features. For individual networks, features constructed using synchronization likelihood (SL) achieved the highest accuracy in distinguishing between cognitive and visual distraction. The optimal feature set from 3 network combinations achieves an accuracy of 95.1% for binary classification and 88.3% for ternary classification of normal, cognitively distracted, and visually distracted driving states. The proposed method could accomplish the augmented recognition of distracted driving states and may serve as a valuable tool for further optimizing driver assistance systems with distraction control strategies, as well as a reference for future research on the brain–computer interface in autonomous driving.

## Introduction

Distracted driving refers to a situation in which a driver’s attention is diverted away from the task of driving due to events, activities, objects, or people inside or outside the vehicle, which leads to delayed confirmation of essential safety-related information for safe driving [1]. Based on the multiple resource theory (MRT) [2], a variety of distraction types can be defined, whereas driving relies heavily on visual perception and cognitive processing. Thus, the 2 most commonly studied types of distractions during driving are visual distraction, also known as “eye-off-road” distraction, and cognitive distraction, referred to as “mind-off-road” distraction [3]. Visual distraction primarily involves external stimuli such as looking at mobile phone, and cognitive distraction engages an internal process when the driver’s mind is occupied with nondriving-related thoughts [4]. Both of these types of distractions can greatly undermine driver’s attention and performance [5,6], albeit through different mechanisms. Facing the multifarious secondary tasks during driving, visual distraction may prevail in certain instances, while in other cases, cognitive distraction may be the dominant factor. Precise identification of the different types of distractions can contribute to the implementation of more oriented prevention measures and strategies. Therefore, gaining a better understanding of driver’s state during moments of distraction and further recognizing distraction types could contribute to reducing the risk of traffic accidents [7,8] and providing the reliable knowledge for supporting advanced intelligent driving systems [9].

Distracted driving state fundamentally involves the driver shifting a portion of their attention from the driving task to a secondary task, resulting in an increase in cognitive workload [10,11]. Distracted driving interferes with driving behavior and impairs driving performance, which is typically reflected in increased reaction times [12,13], instability in speed control [14], and increased variability in lateral control [15]. These changes in driving behavior always occur after a specific time interval following the onset of driving distraction. Therefore, to early identify distracted driving behaviors, there have been a large number of attempts to track the head and eye movement of drivers [16–19]. However, the lack of direct and objective measures in recognizing distracted state is still challenging. Unlike physical indicators, such as eye movements or body posture, which can indicate visual distraction, internal cognitive distracted states are not easily observable. This makes it difficult to accurately assess and differentiate the cognitive distraction states from visual distraction states.

Biomedical signal-based measurements have proven to be effective in the study of driver cognitive status and brain activity, especially the detection of electroencephalogram (EEG), which are the most widely used technique and the most predictive indicator of electrical activity in the brain [20,21]. Compared to the recognition models based on driving variables and eye movements, EEG-based recognition model possesses the capability to provide a deeper understanding of driver cognition by capturing high-resolution brain electrical activity. Martínez et al. [22] proposed an automated framework incorporating channel-based EEG features for detecting driver distraction and validated its efficacy. Ronca et al. [23] developed an EEG-based distraction index by quantifying the driver’s mental workload and attention using the average power of different bands and channels. Considering the intricate interactions among different brain regions with distinct structures and functions, much attention has also been devoted to investigating brain networks [24,25]. Multi-channel EEG signals have been demonstrated to exhibit evident interchannel couplings and rhythmic dependencies. EEG-based functional connectivity maps have shown promising performance in measuring the correlation and statistical dependencies of functional activities across different brain regions [26].

By exploiting the complex functional relationships between different brain regions, complex network theory has been introduced into EEG signal analysis to recognize cognitive state [27]. Rubinov and Sporns [28] described a series of measurement methods for quantifying local and global properties of complex brain networks. They have also designed and developed relevant brain connectivity toolboxes for exploring network attributes of complex structural and functional datasets. Daneshia et al. [29] utilized brain network analysis to identify which regions of the brain are functionally connected to support overtaking in a simulated driving task. Han et al. [30] analyzed brain complex network characteristics during fatigue driving by examining the evolution of EEG signal rhythms. In such research paradigm, graph theory provides a valuable framework for characterizing the organization of functional and anatomical connections in the brain, with the topological properties of networks reflected by metrics [31].

Although the brain electrodes were consistently selected as network nodes, the utilization of various approaches to determine edges could result in diverse brain network topologies [25]. For example, the interaction between brain channels can be quantified using multiple indicators, including various linear and nonlinear synchronization measures based on EEG signals. Linear and nonlinear measurements have both proven to be valuable, with each indicator having its own advantages and limitations [32–34]. However, relatively few studies have evaluated the performance of different synchronization indicators of brain network in distracted state recognition scenarios. Perera et al. [35] endeavored to classify distracted and nondistracted driving tasks using brain connectivity estimators, but fell short in achieving a deeper differentiation between visual distraction and cognitive distraction.

The aim of this study was to establish an augmented recognition framework of distracted driving states by leveraging varied synchronization indicators in brain networks. A simulated car-following experiment containing 4 distraction subtasks was designed to encompass the cognitive distraction and visual distraction states. Three connectivity indices including synchronization likelihood (SL), phase locking value (PLV), and coherence indicator were selected to construct functional brain networks. The connectivity strength as well as 4 global topological features were calculated to explore the potential relationship between the configuration of the brain network and the occurrence of driving distraction. Subsequently, the machine learning classifiers were trained and implemented to recognize the different distracted driving states based on brain network features.

The main contributions of the paper are listed as follows:

a. The configuration of the functional brain network during distracted driving is constructed through electrophysiological analysis using 3 synchronization indicators as network edges and 4 global topological features as network properties.

b. The performance of different synchronization indicators in brain networks is compared and the SL presents optimal recognition capability in distinguishing between normal and distracted driving states using single brain network knowledge.

c. The augmented framework of recognizing normal, visual distraction, and cognitive distraction states is proposed, and the best classification performance is achieved by utilizing the combined global topological features of the 3 varied brain networks characterized by different synchronization indicators.

The following sections of this paper are structured as follows: the “Materials and Methods” section outlines the materials and methods of this study; the “Results” section presents the results of task load, brain network analysis, and classification outcomes; the “Discussion” section delves into the discussion; and the “Conclusion” section provides the conclusion.

## Materials and Methods

### Experimental design

#### Driving simulation experiment

In the simulated scenario, the experimental road consisted of an 8-km-long, 2-way, 4-lane road without any intersections and with a speed limit of 80 km/h to simulate the car-following behavior in free-flow traffic on urban freeways. There are primary task and secondary task in the setting of the driving simulation experiment.

1. Primary task

The main task of the participants was to drive the vehicle and follow the leading vehicle in front, without overtaking, and to maintain an appropriate following distance throughout the experiment. The participants were instructed to drive in the innermost lane, with 2 leading vehicles positioned on the 2 inner lanes in front. To account for the following ability of the vehicles in the simulated driving environment, as well as the actual traffic speed on the road, the speed of the leading vehicles was set at 60 km/h. Moreover, to better simulate the actual following scenario and reduce the learning effect, the speed of the leading vehicles was subject to small fluctuations within a certain range to mimic the random acceleration and deceleration of actual vehicles [36].

2. Secondary task

**Fig. 1.**
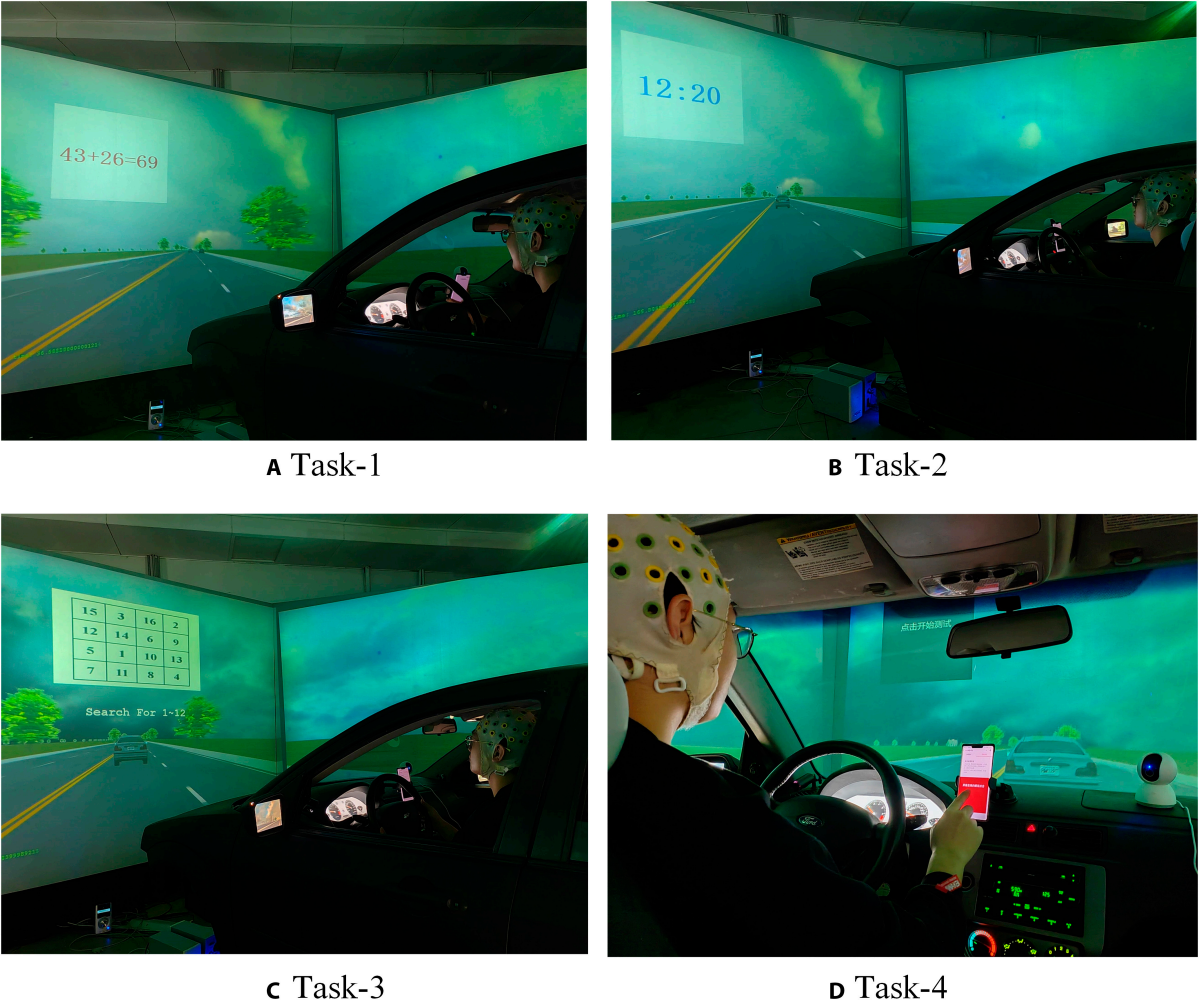
Secondary driving tasks in the simulated experiment.

In addition to performing the primary task of following the leading vehicle during driving, the participants were also instructed to perform 4 different distracted secondary tasks after a certain period of following time. Four different tasks were designed to simulate the distracted driving behavior that can occur during actual driving due to various factors. The scenarios of secondary tasks in the driving simulator are shown in Fig. 1. The 4 subtasks include math calculation task (task-1), clock angle discrimination task (task-2), number sequence recognition task (task-3), and mobile phone task (task-4). Based on the replicability and controllability of driving scenarios in the driving simulator, each secondary driving task was repeated 5 times sequentially from task-1 to task-4. Following the completion of each task type, drivers engaged in a period of steady-state car-following tasks before proceeding to the next task type. There was a minimum interval of 15 s between each subtask to allow drivers to adjust their state and alleviate fatigue during visual tasks. Feedback buttons were provided for each task, and feedback on secondary tasks was not required if stable car-following status could not be maintained.

The first secondary task involved math problems that required the participants to judge their correctness. The problem content was addition and subtraction of 2-digit numbers, and the participants’ responses of correctness were collected through in-vehicle response devices. The second distracted secondary task required the participants to perform a clock angle discrimination task, in which a random time was given and the participants were required to judge whether the angle formed by the hour and minute hands was greater than 90° based on the instructions given before the experiment. Similar to the first one, it also demanded the participants’ focused attention and cognitive processing. To accentuate cognitive distractions, the picture prompts for the mathematical calculation problem and the clock angle identification problem were presented in a flashing manner. This approach ensures that the secondary task pictures do not capture the driver’s visual attention, thereby allowing only cognitive distractions to remain once the task pictures disappear.

The third task involved a high visual workload, requiring the participants to identify a reasonable sequence of numbers. This task simulates the distracted behavior that may occur when drivers are attracted by advertisements or other signs on the road during real-world driving [37]. The fourth secondary driving task involved a color-changing task on a mobile phone. The participants were required to continuously watch the phone and click the feedback button on the screen when a specific color appeared. The phone was already pre-set to the task screen and placed on the right side of the driver’s seat. The task picture in task-3 and color screen of phone in task-4 are consistently displayed for a duration of 10 s to induce visual distraction of drivers. In contrast to task-1 and task-2, task-3 and task-4 require lower cognitive load but greater visual engagement due to eye tracking and glancing. The details of different types of distraction subtasks are given in Table 1. In this study, we labeled task-1 and task-2 as cognitive distraction, and task-3 and task-4 as visual distraction according to their dominated types.

**Table 1.** Types of distraction subtasks

Task ID	Subtask	Dominated type	Task form
1	Math calculation	Cognitive	Decision
2	Clock angle discrimination	Cognitive	Mental
3	Number sequence recognition	Visual	Visual-tracking
4	Mobile phone task	Visual	Visual-attention

After arriving at the experimental site, all participants read and signed an informed consent form and completed some basic questions related to distracted driving on the road. Then, they were introduced to some requirements of the driving experiment, and the mobile phone in secondary task 4 was placed in advance. Each participant underwent about 10 min of driving practice in the simulator and learned about the feedback method of the secondary task. During the formal experiment, each participant completed about 10 min of simulated driving while following a leading vehicle, and completed the designated secondary driving task during the process. The experimental procedures were approved by the Science, Technology, and Academic Ethics Committee of the School of Traffic and Transportation of Beijing Jiaotong University.

#### NASA task load index

After each driving task, the workload was evaluated using the NASA Task Load Index (NASA-TLX) questionnaire according to their performance in different driving conditions [38]. NASA-TLX is a validated measurement method for assessing subjective state workload, and it has been used in many distracted driving studies to initially assess the difference in driving workload between normal and distracted driving states.

### Participants

This study recruited 36 healthy participants with driver’s licenses, including 24 males (mean age = 23.9 years, SD = 1.72) and 12 females (mean age = 23.3 years, SD = 0.89), most of whom were college and graduate students. All participants had normal vision or corrected-to-normal vision and no history of neurological or brain diseases. All participants had owned legal driving licenses for more than 1 year. Prior to the experiment, a 10-min period was allotted for each participant to become familiar with the driving simulator and adjust their seat to achieve optimal comfort for driving. Participants with a history of simulator sickness were excluded from the study. All participants’ written informed consents were obtained before the experiment.

### Data collection and processing

The EEG data were acquired using a collection device consisting of a 64-channel electrode cap (Brain Products) and bioelectric amplifier system (actiCHamp), with a sampling frequency of 1,000 Hz. The electrode channel positions on the scalp were placed according to the standard of the international 10-10 system. The original EEG data may contain various artifacts, such as blink and line noise, which require preprocessing before analysis. The raw data were processed using the EEGLAB toolbox and MATLAB 2021b. First, the raw data are resampled to 512 Hz to simplify subsequent data processing. Next, a bandpass finite impulse response (FIR) filter is applied to remove noise below 0.5 Hz and above 40 Hz. Bad electrodes are replaced with interpolated adjacent channels. The data undergo re-referencing and baseline correction, and last, the ADJUST plugin is used to remove artifacts including blinks, eye movements, and general discontinuities, resulting in clean EEG data for analysis [39]. The preprocessed data were then partitioned into epochs based on task definitions.

### Complex network construction

Functional connections and states of brain regions during driving undergo transformations, and we attempt to identify such changes from a complex network perspective. Nodes and edges are fundamental elements that constitute a complex network. For EEG data with 63 active channels corresponding to the number of nodes, the existence of an edge between any 2 nodes depends on the functional connections between the 63-channel EEG signals. In the field of EEG, researchers have proposed various algorithms to measure the correlation between 2 signals [32], which is referred to as functional connectivity metrics. Prior to computing the functional connectivity metrics, the EEG signals were decomposed into 4 subbands via wavelet packet transformation: δ-band (0.5 to 4 Hz), θ-band (4 to 8 Hz), α-band (8 to 13 Hz), and β-band (13 to 30 Hz), and the corresponding connectivity matrices were computed for each subband.

#### Synchronization likelihood

The likelihood synchronization algorithm in generalized synchronization is effective for processing nonstationary signals, and the SL can measure the nonlinear synchronization between each pair of EEG signals according to Eq. 1. It provides a simple normalized estimate of the mutual dependence between 2 or more simultaneously recorded time series signals, and gives more accurate information about functional interactions in dynamical systems [40]. The range of SL values is between 0 and 1, where 0 indicates complete asynchronization between the 2 signals and 1 indicates complete synchronization, although these extremes are rarely encountered in actual signals.

$$\begin{array}{c} {\mathrm{SL}}_{\mathrm{xy}}=\frac{1}{N}\sum_{t=1}^{N}\left(\frac{1}{2\left(w_{2}-w_{1}\right)}\sum_{\begin{array}{c} \;\tau =1\\ w_{1}<\left|t-\tau \right|<w_{2} \end{array}}^{M}{\mathrm{SL}}_{\mathrm{xy}}\left(\tau \right)\right) \end{array}$$ (1)

where SL*_xy_* describes the SL value between channel *x* signal and channel *y* signal; *w*_1_ and *w*_2_ denote 2 time windows; *N* and *M* denote the number of sampling points; SL*_xy_*(*τ*) is the SL value at moment *τ* between channel *x* signal and channel *y* signal.

#### Phase locking value

PLV is a time-domain measurement of the phase synchronization information, which measures the phase difference between 2 channel signals. The synchrony brain network constructed using PLV is only related to the phase of the signals, ignoring the differences in signal amplitudes [41]. The range of PLV values is [0, 1], where a higher value indicates a stronger phase synchronization between 2 signals. However, PLV is sensitive to volume conduction effects. The PLV value is calculated according to Eq. 2.

$$\begin{array}{c} {\mathrm{PLV}}_{\mathrm{xy}}=\frac{1}{N}\left|\sum_{t=1}^{N}\text{exp}\left(j\left(\varphi_{x}\left(t\right)-\varphi_{y}\left(t\right)\right)\right)\right| \end{array}$$ (2)

where *φ_x_*(*t*) and *φ_y_*(*t*) are the phases of the channel *x* signal and channel *y* signal, respectively, at the moment *t*; *j* is the unit of an imaginary number; *N* represents the number of time points; PLV takes the value in the range of [0 1], with a larger value indicating a stronger degree of phase synchronization between the 2 signals.

#### Coherence

Intercorrelations between EEG signals can be characterized by calculating coherence (COH). Coherence can be regarded as a quantitative measure of the correlation of 2 signals in the frequency domain, which can be used to evaluate the linear correlation between 2 channels. The coherence coefficient is a normalized quantity bounded by 0 and 1, with larger values indicating that the 2 signals are more correlated [29]. The calculation of coherence is as follows in Eq. 3.

$${\mathrm{COH}}_{\mathrm{xy}}=\frac{1}{F}\sum_{f=1}^{F}{\left|K_{\mathrm{xy}}\left(f\right)\right|}^{2}$$ (3)

where *F* represents the size of the frequency domain; *K_xy_*(*f*) is a coherent function of the frequency f between channel *x* and channel *y*, and denotes the ratio between the cross-spectrum power density and the individual spectral power density of the 2 signals as shown in Eq. 4.

$$K_{\mathrm{xy}}\left(f\right)=\frac{S_{\mathrm{xy}}\left(f\right)}{\sqrt{S_{\mathrm{xx}}\left(f\right)S_{\mathrm{yy}}\left(f\right)}}$$ (4)

where *S_xy_*(*f*), S*_xx_*(*f*), and S*_yy_*(*f*) are the expressions for their respective power spectral densities between channel *x* and channel *y*.

### Brain network topology analysis

Theoretically, networks are typically represented using graphs. Using graph theory, brainwave information is simplified into a collection of vertices and weighted edges, where their interaction information or connectivity reflects the importance or strength of certain interactions between 2 vertices [28]. The matrix coefficient is denoted as C*_xy_*, connecting channel *x* and channel *y*, and the value could be defined by the 3 functional connectivity measurement indicators mentioned above, SL*_xy_*, PLV*_xy_*, and COH*_xy_*. Next, the obtained connectivity matrix is filtered in order to transform it into a graph. The so-called “filter” refers to removing spurious connections in the obtained connectivity matrix and retaining only the connections that we consider to be genuine.

Removing spurious connections of the connectivity matrix refers to reconstructing the graph by selecting an appropriate threshold. To effectively address the variability of different connectivity metrics, we apply the sparsity thresholds to all connectivity matrices, which are calculated by the ratio of reserved functional connections to the total number of possible connections [42]. There are 2 principles for selecting appropriate sparsity thresholds: first, that the average degree of all nodes in each filtered network is greater than 2log(*n*), where *n* is 63 in this study, denoting the number of nodes, and, second, that the small-world scalars of the filtered networks are all greater than 1.1. Accordingly, the sparsity values ranging from 13% to 47% in steps of 1% are selected. The connectivity matrices are converted using the thresholds to binary adjacency matrices. Finally, using graph analysis on the binary adjacency matrices, the topological features of brain networks can be exported.

To quantitatively analyze the differences in brain network topology between the distracted state and the normal state, we conducted graph theory analysis using the Brain Connectivity Toolbox. An essential component of any graph theoretical analysis is comparing the metrics obtained from the empirical network to the network ensemble appropriately configured to represent the null hypothesis [28]. For the brain networks constructed under different sparsity thresholds, we selected 4 topological features to characterize the networks: characteristic path length (L*_g_*), clustering coefficient (C*_g_*), global efficiency (E*_g_*), and local efficiency (E*_l_*). These spatial pattern of network (SPN) features are expected to provide the spatial organization information and patterns of connectivity within the network.

The characteristic path length [43] is the average distance between all nodes in the network, which reflects the connectivity of the network, defined as Eq. 5.

$$L_{g}=\frac{2\sum_{x\geq y}L_{\mathrm{xy}}}{n\left(n-1\right)}$$ (5)

where *n* represents the number of nodes and L*_xy_* denotes the length of the shortest path between channel *x* and channel *y*. L*_g_* is an indicator of the overall routing efficiency of the network.

The clustering coefficient [42] can indicate the likelihood that the neighbors of a vertex are also connected to each other, reflecting the degree of modularization and functional differentiation in the brain functional network, defined as Eq. 6.

$$C_{g}=\frac{1}{n}\sum_{x\in n}\frac{2e_{x}}{k_{x}\left(k_{x}-1\right)}$$ (6)

where *k_x_* is the total number of neighboring channels of channel *x* in the network and *e_x_* is the total number of actual connected edges between channel *x* and its neighboring channels. C*_g_* is the probability that the neighbors of a vertex are also connected to each other, which is the arithmetic mean of the clustering coefficients of all nodes in the network.

The global efficiency [28] is used to measure the capacity of brain functional networks to transmit and process information, defined as the average inverse shortest path length in Eq. 7.

$$E_{g}=\frac{1}{n\left(n-1\right)}\sum_{x\neq y}\frac{1}{L_{\mathrm{xy}}}$$ (7)

where L*_xy_* is the shortest path length between channel *x* and channel *y*. The global efficiency measures the efficiency of local information transmission and processing.

The local efficiency [28] is then defined as in Eq. 8.

$$E_{l}=\frac{1}{n}\sum_{x\in n}E_{g}\left(G_{x}\right)$$ (8)

where G*_x_* refers to the subgraph composed of the neighbors of channel *x* and E*_g_*(·) computes the global efficiency of the subgraph.

Since these features are not computed at a particular sparsity, but rather a selected sparsity range space, the average values of the aforementioned features are characterized by calculating the area under the curve (AUC) for each filtered matrix [44,45].

### Classification algorithm

Based on different metric characteristics of the network and their corresponding complex network features, we attempted to classify different driver states (normal, cognitively distracted, and visually distracted driving states) using varied network combinations. Considering the performance, complexity, and flexibility of classifiers, it is useful to test and compare various classification methods. Building upon prior EEG classification studies [46,47], 4 methods were selected for comparison: distance-based K-nearest neighbor classifier (KNN), Random Forest (RF), eXtreme Gradient Boosting (XGBoost), and Support Vector Machine (SVM). Notably, all these models were trained through 10-fold cross-validation, reserving 10% of the data for testing. The KNN model was configured as a moderate KNN with Euclidean distance as the measurement method. Given the relatively low feature count, SVM utilized a Gaussian function as the kernel function, and data were standardized during feature input.

### Statistical analysis

Analysis of variance (ANOVA) is used to evaluate NASA-TLX, aiming to preliminarily examine the workload difference between distracted driving state and normal driving state throughout the entire experiment. Additionally, to reflect the differences in the brain topological networks between 2 driving states (distracted and normal driving), ANOVA was used to compare the values of SL, PLV, COH, and complex network features separately for each frequency band. To ensure the reliability and accuracy of the results, multiple comparison correction methods were applied in the statistical process. Significance values were adjusted for multiple tests by Bonferroni correction, and all appearing *P* values in the text are corrected *P* values. The significance level for all the statistical analyses mentioned above was set at 0.05, and the analyses were conducted using SPSS for Windows.

## Results

### NASA-TLX analysis

**Fig. 2.**
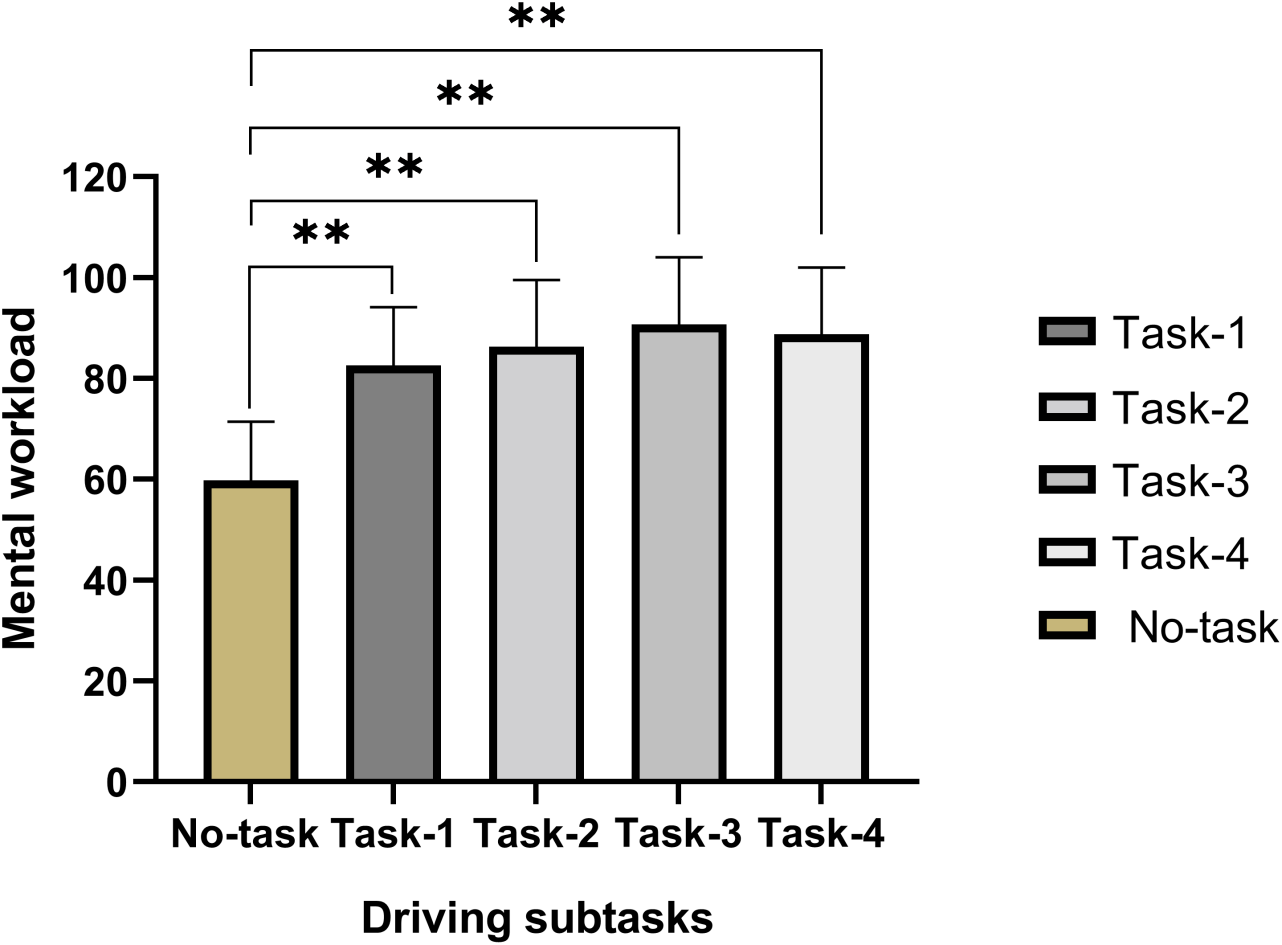
Driving workload under different subtasks. No-task, normal driving; task-1, math calculation task; task-2, clock angle discrimination task; task-3, number sequence recognition task; task-4, mobile phone task.

The NASA-TLX scores of participants performing different driving tasks are shown in Fig. 2, reflecting the driving workload. Statistical analysis of the questionnaire results indicated that the mental workload of participants increased when engaging in driving subtasks, with a significant impact on mental workload (*F* = 67.105, *P* = 0.00 < 0.01). Compared to unmanned secondary task conditions, there is a significant increase in driver workload when performing this task, with minimal variations among subtasks. It can be preliminarily inferred that the participants were distracted while driving due to the interference of the driving subtasks, as they needed to manage the other subtasks while maintaining a reasonable following distance. Comprehensively considering the task design and workload level, the distraction states were categorized into cognitive distraction including the math calculation task (task-1) and clock angle discrimination task (task-2), and visual distraction including the visually demanding number sequence recognition task (task-3) and mobile phone task (task-4).

### Driving behavior analysis

Kruskal–Wallis tests were performed on the longitudinal and lateral driving performance metrics in the delineated states. As shown in Table 2, the driving behaviors are affected while performing the distraction subtask. The significant speed difference is only observed between the cognitive distraction (C) and normal driving (N). Furthermore, the variations in the centerline offset between visual distraction (V) and normal driving (N) state are significant, highlighting the compensatory behavior of drivers as they rectify momentary lapses in visual attention.

**Table 2.** Driving performance under different driving states

Driving performance	N	C	V	*P* (C vs. N)	*P* (V vs. N)	*P* (C vs. V)
Speed (m/s)	16.35 ± 0.73	15.82 ± 0.69	16.12 ± 0.69	**0.00**	0.78	0.57
Acceleration (m/s^2^)	−0.19 ± 0.25	0.03 ± 0.37	0.05 ± 0.39	**0.00**	**0.00**	1.00
Centerline offset std (m)	0.22 ± 0.10	0.22 ± 0.09	0.28 ± 0.12	1.00	**0.001**	**0.004**
Steering angle std (°)	0.029 ± 0.02	0.024 ± 0.01	0.030 ± 0.02	0.69	0.11	**0.01**

Bold text represents significant results (*P* < 0.05). N, normal driving; C, cognitive distraction; V, visual distraction; std, standard deviation.

### Network analysis

**Fig. 3.**
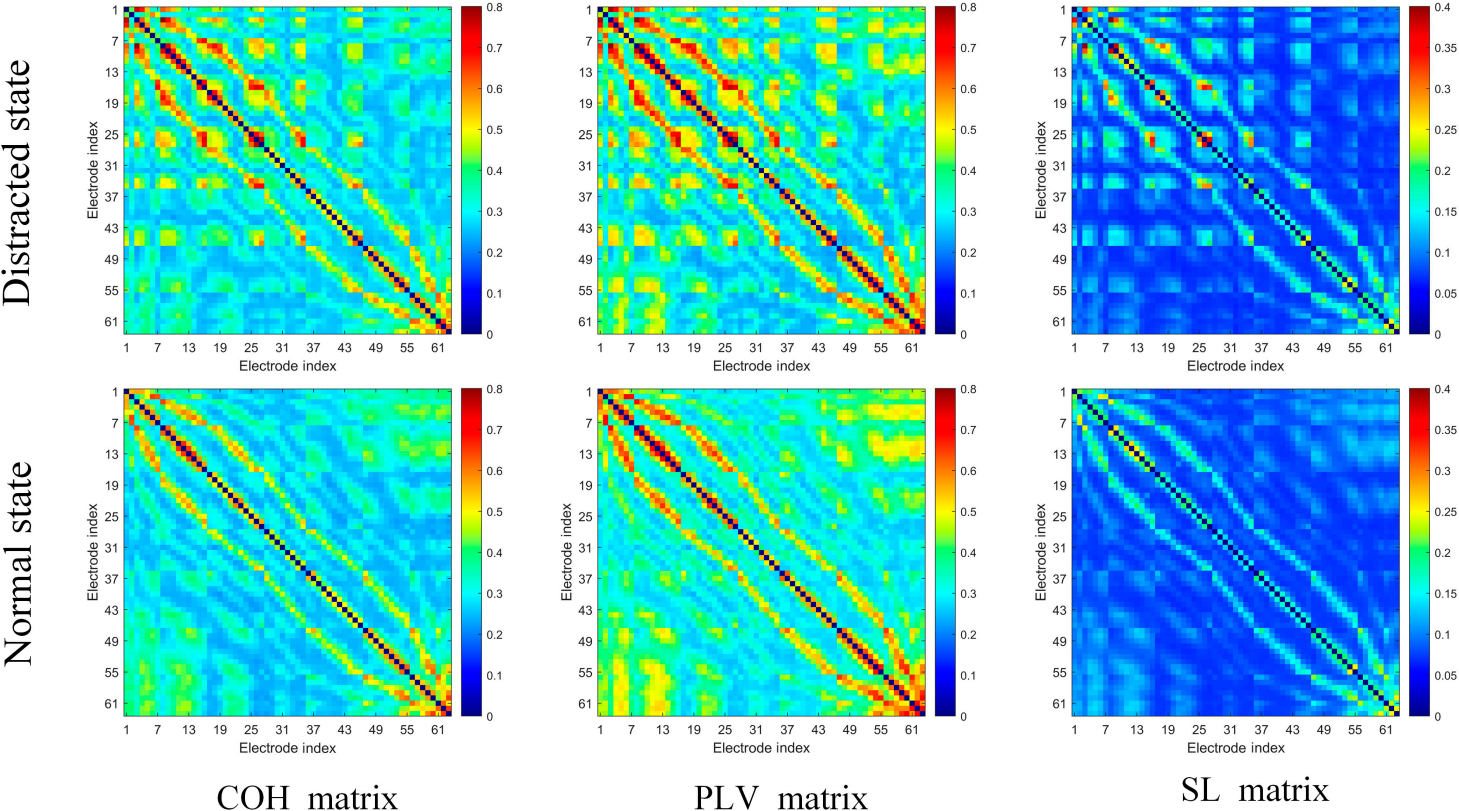
Comparison of connectivity matrices between distracted state (top) and normal driving state (bottom), with matrices shown from left to right: the COH matrix, PLV matrix, and SL matrix. The matrix is a 63 × 63 square matrix, where *x* and *y* axes correspond with the channel numbers. The connection strengths are indicated by a color scale from blue to red.

The comparison of average strength matrices under distracted and nondistracted states for the 3 connectivity indices, namely, COH, PLV, and SL, is illustrated in Fig. 3, where each state is obtained by averaging all positive connections across participants. While variations in strength are observed among different connectivity matrices, the strength matrices of the 3 connectivity indices exhibit complex yet similar structures. In vertical comparison, the strength under the distracted state shows higher activity than the normal driving state, with a noticeably denser network structure. However, this increased activity is regional in nature, and on average, the connectivity strength exhibits moderate statistical correlation (median comparison in PLV: 0.333/0.336; COH: 0.306/0.291; SL: 0.085/0.085). In horizontal comparison, the network formed by SL connections appears relatively sparse, with only a few instances of high synchronization values, and intermittent synchronization behavior is observed between regions. In contrast, PLV and COH demonstrate broader and higher level of synchronization patterns.

Variance analysis was employed to assess the differences in connectivity between normal, cognitive distraction, and visual distraction states across each frequency band. Table 3 presents the results of the comparison of the 3 connectivity measures, which show that although the 3 connectivity measures are calculated in different ways, they exhibit significant differences in functional connectivity strength for both states, especially in the θ and β bands. Consistently, in the θ frequency band, the connectivity strength under the distracted state was significantly higher compared to the normal state, for mean SL (*P* < 0.01), mean PLV (*P* < 0.01), and mean COH (*P* < 0.01). Similarly, this difference was observed in the β frequency band, where the connectivity strength was higher under the distracted state, for mean SL (*P* < 0.01), mean PLV (*P* < 0.017), and mean COH (*P* < 0.01). Furthermore, except for the network constructed using SL, the networks based on COH and PLV as connectivity measures also exhibited differences in the remaining 2 frequency bands. In the α frequency band, the connectivity strength under the distracted state was significantly different from that under the normal state, for mean PLV (*P* < 0.01) and mean COH (*P* < 0.01).

**Table 3.** The connectivity strength under different driving states

Connection metrics	Frequency band	Normal	Cognitive distraction	Visual distraction	*P* value
SL	Delta band	**0.125**	**0.124**	**0.131**	**0.036**
	Theta band	**0.085**	**0.086**	**0.097**	**<0.01**
	Alpha band	0.090	0.088	0.087	0.175
	Beta band	**0.077**	**0.088**	**0.078**	**<0.01**
PLV	Delta band	**0.369**	**0.386**	**0.383**	**<0.01**
	Theta band	**0.374**	**0.389**	**0.390**	**<0.01**
	Alpha band	**0.412**	**0.469**	**0.374**	**<0.01**
	Beta band	**0.283**	**0.292**	**0.291**	**0.017**
COH	Delta band	**0.348**	**0.416**	**0.374**	**<0.01**
	Theta band	**0.310**	**0.344**	**0.342**	**<0.01**
	Alpha band	**0.328**	**0.315**	**0.312**	**<0.01**
	Beta band	**0.275**	**0.297**	**0.289**	**<0.01**

Bold text represents significant results (*P* < 0.05).

### Topological feature analysis

**Fig. 4.**
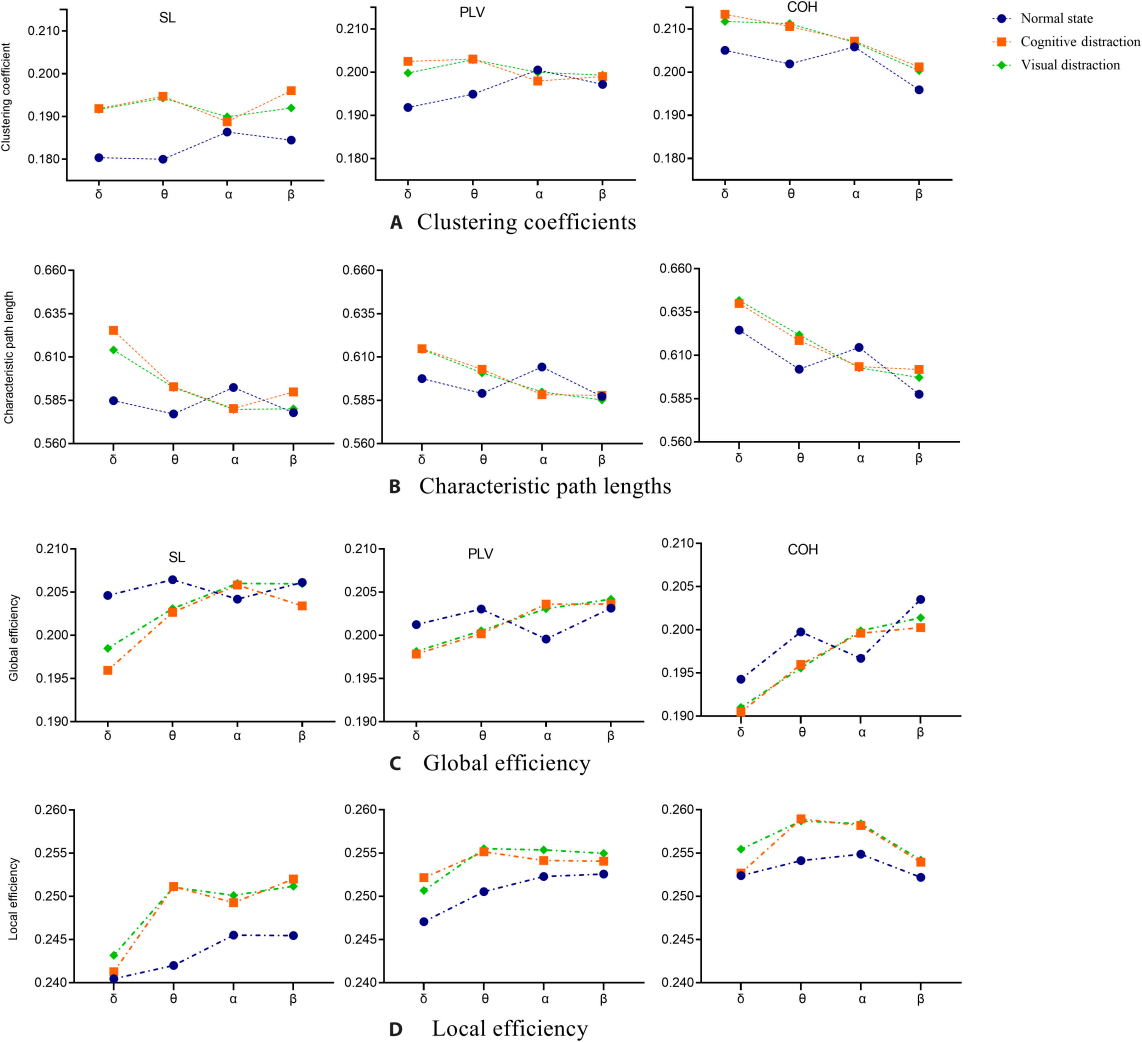
Network structure features for 3 driving states: (A) clustering coefficients, (B) characteristic path lengths, (C) global efficiency, and (D) local efficiency, with feature value magnitudes on the *y* axis and frequency bands on the *x* axis. Left to right corresponds to the networks SL, PLV, and COH.

After constructing functional connectivity networks using various connectivity metrics, quantitative analysis based on graph theory was performed to investigate the topological properties of the networks under distracted and normal driving states. Graph theory is a mathematical technique utilized to understand the topological characteristics of complex brain networks. Network structure and efficiency characteristics were calculated for each network, the former including clustering coefficients and characteristic path lengths, and the latter including global and local efficiencies. The network structural values reported in Fig. 4 A and B highlight that, for all partitions, the 3 network structures exhibit similar performance. In the distracted state, the network exhibited higher clustering coefficients in the θ band, with the most significant difference compared to the normal driving state (*P* < 0.01). In general, higher clustering coefficients indicate that nodes in the network tend to form highly connected local clusters. In terms of path length, the 3 networks showed similar patterns of variation, most of which could help to distinguish the normal and distracted states. However, the difference of the states was not significant in the β-band (*P* = 0.710) for the PLV network. Overall, the normal driving state differed from the cognitively distracted and visually distracted driving states in most of the frequency bands, but for the distinction between the 2 distracted states, the variability was only evident in the δ and β part of the frequency band.

The network efficiency values include global efficiency and local efficiency, which are scalar measures of information flow. As shown in Fig. 4 C and D, consistently, all networks in the distracted state showed higher local efficiency values and the local efficiency values all showed peaks in the theta band, which differed most from normal driving, as reported by the results of the ANOVA (SL: *F* = 113.2, *P* < 0.01; PLV: *F* = 44.4, *P* < 0.01; COH: *F* = 31.6, *P* < 0.01). As for the global efficiency, the global efficiency in the 2 distraction states showed an increasing trend among the 4 frequency bands, and only in the α-band the global efficiency value was higher than that of the normal driving state. The above combined indexes suggest that the presence of secondary tasks affects the functional organization of the driver’s brain to undergo adaptive changes.

### Classification results

In order to validate the effectiveness of the different synchronization measures of the networks and the complex network features, we used the values of connection strength V and topological features (clustering coefficients C*_g_*, characteristic path lengths L*_g_*, global efficiency E*_g_*, and local efficiency E*_l_*) under 4 frequency bands as inputs (i.e., 3 networks × 4 frequency bands × 5 network features) for the model validation of the dichotomy between normal and distracted driving states. The performance analyses of the different classifiers are presented in Table 4. The selected classifiers were evaluated based on accuracy (Acc), precision (P), recall (Re), F score (F1), and AUC. For the binary classification model, XGBoost demonstrates superior adaptability, outperforming other classifiers in all performance aspects. It achieves the highest accuracy of 95.1% with an AUC value of 0.98. In subsequent discussions, the XGBoost model was selected for the evaluation of multi-state outcomes and feature analysis.

**Table 4.** Binary classification performance of different classifiers

Normal and distracted driving state					
Models	Acc (%)	P (%)	Re (%)	F1	AUC
SVM	92.2	92.1	92.1	0.92	0.92
RF	87.2	87.6	87.3	0.87	0.87
XGBoost	95.1	98.0	92.5	0.95	0.98
KNN	85.1	86.1	84.2	0.85	0.92

**Fig. 5.**
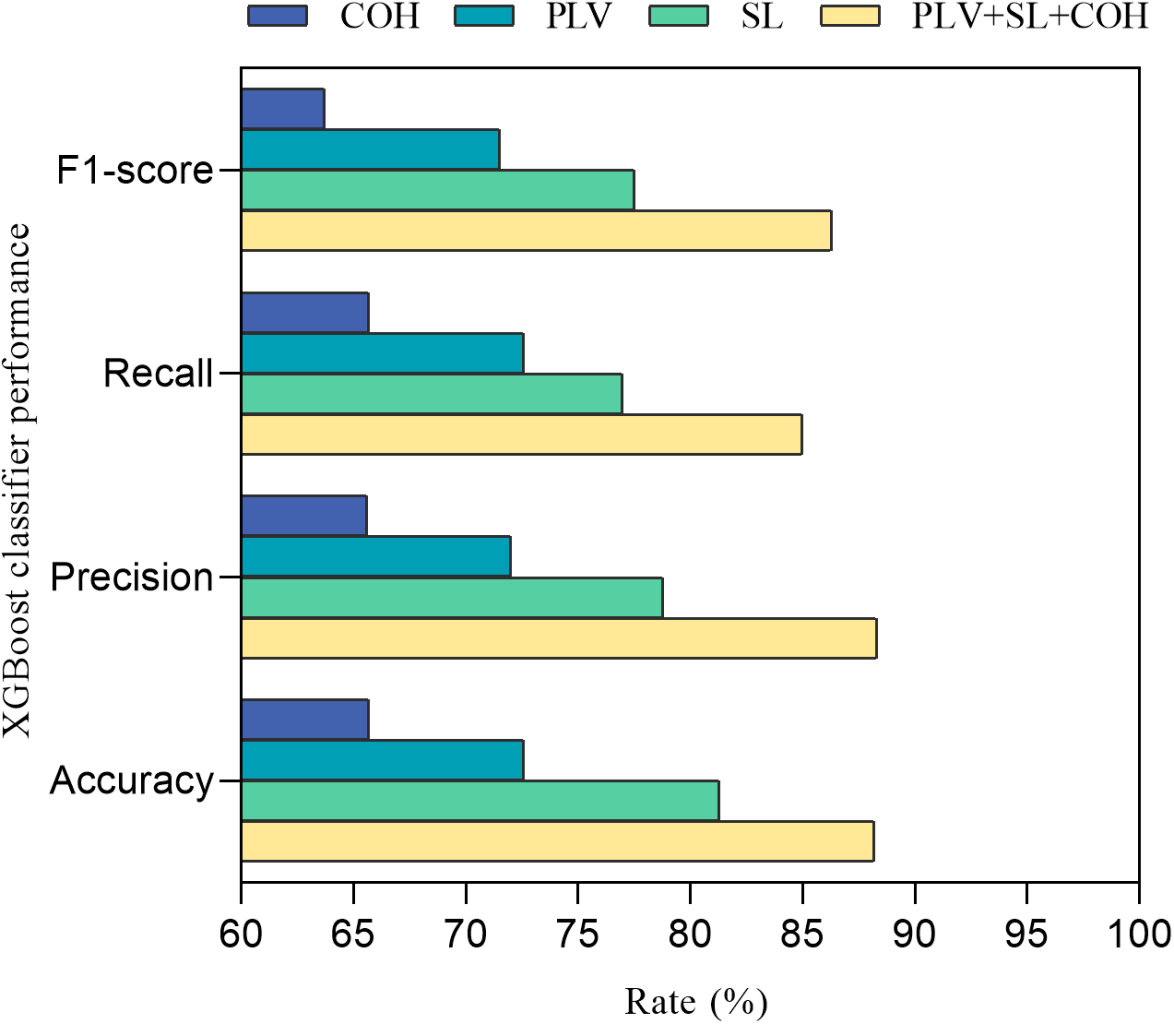
XGBoost classifier performance with different feature sets.

**Fig. 6.**
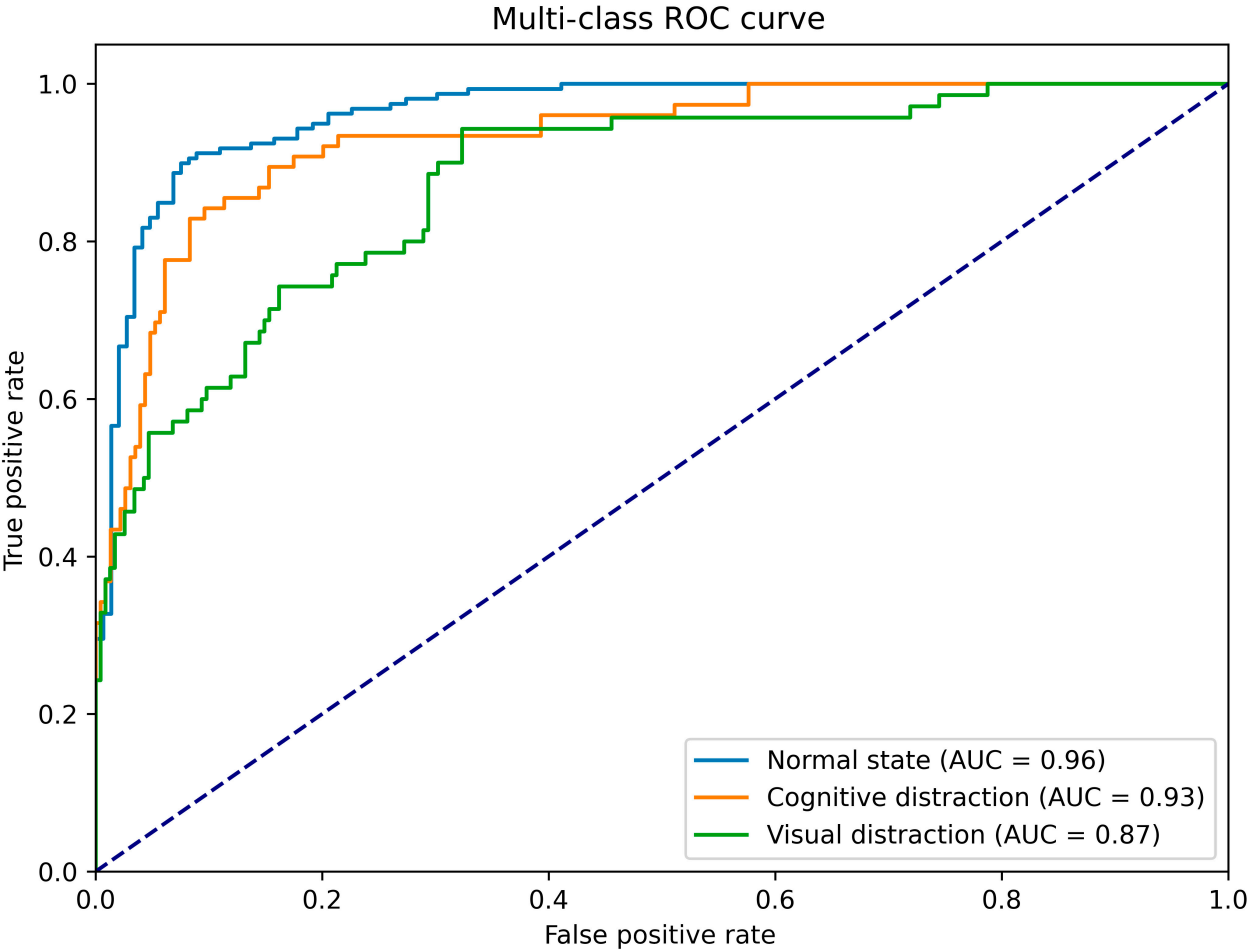
ROC curves of the XGBoost classifier for the optimal set of features (combination of SL, PLV, and COH network features).

Based on the above assessment of the performance of the selected classifiers for binary classification, the XGBoost model was selected for feature combination of the network features to classify and identify the 2 different distraction states, visual and cognition. The same 10-fold cross-validation was used to tune the model. As shown in Fig. 5, for normal, cognitively distracted, and visually distracted driving states, the SL network performs the best out of the 3 networks, with accuracy, precision, recall, and F1 scores that can reach 81.3%, 78.8%, 77%, and 0.78. The performance of feature combination of the 3 networks (PLV + SL + COH) is the best, with the performance of accuracy, precision, recall, and F1 scores improving to 88.2%, 88.3%, 85.0%, and 0.863. The receiver operating characteristic (ROC) curves for state classification are shown in Fig. 6. By observing the ROC graphs and comparing the AUC, the model predicts the highest AUC value of 0.96 for recognizing normal driving states, while the cognitive distraction AUC value was 0.93 and the visual distraction state AUC value was 0.87.

## Discussion

This study constructed multiple functional connectivity networks from electrophysiological signals to investigate whether brain network structure and topological differences can effectively recognize the distracted driving states. The study designed a simulated driving task, inducing driver distraction through different secondary task interferences. From the perspective of driving behavior, visual distraction has been observed to impede lateral control [48], as drivers tend to compensate for the error of taking their eyes off the road. Additionally, high-level cognitive load is often associated with a reduction in average driving speed [49,50]. These behavior differences are consistent with our findings, which reflects the effectiveness of the secondary task paradigm employed in our experiment.

EEG signals were collected to construct brain functional networks for state comparison and augmented recognition. The results showed that SL network features demonstrate superior recognition capability for binary classification of distracted driving states, while the combination of multiple networks yielded the best performance on the multiple classification of normal driving, visual distraction, and cognitive distraction, indicating the complementary nature of different brain networks. Table 5 lists the state-of-the-art (SOTA) works that have examined EEG signals in recognizing driving distracted state, and our proposed method presents higher recognition accuracy.

**Table 5.** Recognition performance compared with SOTA

SOTA works	Method	Classification type	Accuracy
Li et al. [64]	Temporal–spatial deep learning	Binary/multiple	92%/88%
Zuo et al. [65]	VS + MSE	Binary	92.48%
Wang et al. [66]	PSD	Multiple	78.66%
Martínez et al. [22]	Pearson matrix	Binary/multiple	83.9%/73%
Our (2024)	SL + PLV + COH	Binary/multiple	95.1%/88.2%

Moreover, we further investigate the brain cognitive functions affected by distraction, which emerge from the interactions between neural populations across different brain regions [51]. We found that in the alpha band, all connectivity values were slightly lower in the distracted state compared to the normal state, while in the theta and beta frequency bands, they were significantly enhanced, as shown in Table 3. Similar findings were observed in [52], that is, the increased mental workload and task difficulty were associated with elevated theta and decreased alpha functional connectivity. It has been shown that most of the changes related to cognitive task difficulty in functional connectivity analysis occur in frontal theta and beta activities [53].

**Fig. 7.**
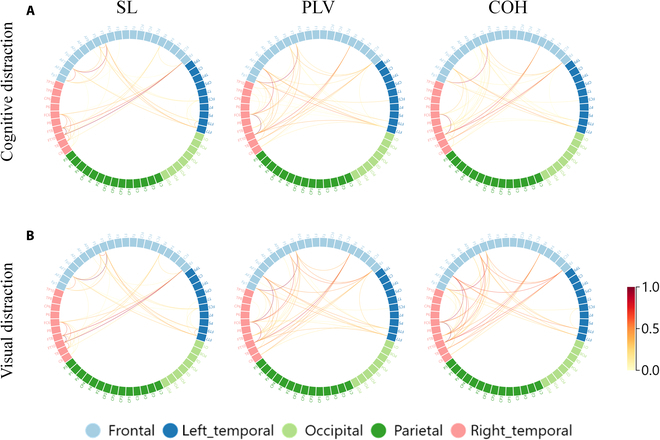
Connectivity strength differences among varied driving states in θ band. Only the top 2% connections with the increased connectivity strengths are preserved. Color bars indicate connectivity strengths with normalized values ranging from 0 to 1; the values indicate the magnitude of change in connectivity strengths between different states.

**Fig. 8.**
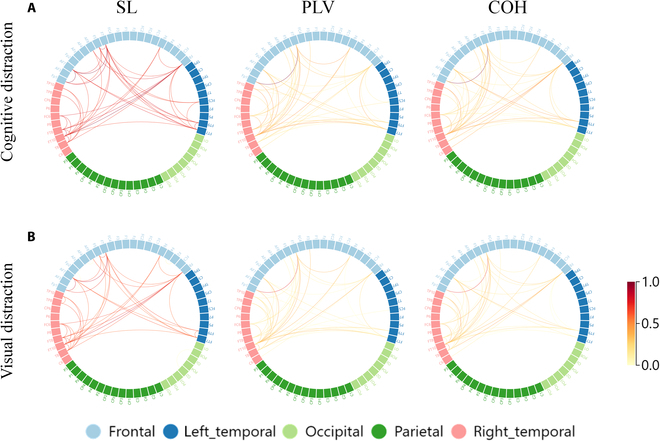
Connectivity strength differences among varied driving states in β band. Only the top 2% connections with the increased connectivity strengths are preserved. Color bars indicate connectivity strengths with normalized values ranging from 0 to 1; the values indicate the magnitude of change in connectivity strengths between different states.

To intuitively characterize the theta and beta band changes in brain connectivity under different states, we plotted connectivity strength chords by subtracting the connectivity strength of the distracted driving states from the normal driving state. For better illustration, we retained only the top 2% of connectivity strength changes for the increasing connectivity. Figure 7 illustrates the changes in connectivity patterns in the theta band. In the distracted state, as secondary tasks appeared, drivers reported an increased workload as they focused on one of the tasks, leading to an enhancement effect in the frontal lobes and related regions. This is evidenced in Fig. 7 A and B by an increase in connectivity within the frontal (F), central frontal (FC), and frontotemporal (FT) lobe regions, manifesting a more pronounced enhancement effect for the visual distraction in COH network. Supporting our findings, the researches on brain region activation reveal the consistent localization of activity in the frontal lobe during distraction [54,55]. Similarly, Fig. 8 illustrates changes in connectivity patterns in the beta band, with increasing connectivity strength under both distracted states. Unlike the theta band, the connections were stronger under cognitive distraction, as frontal beta signals could be more easily modulated by cognitive tasks [56]. Furthermore, the connectivity patterns of different synchronization measures (SL, PLV, and COH) appear to exhibit a commonality, with greater involvement of the frontal, left temporal, and right temporal lobes compared to other regions in the distracted state [57].

**Fig. 9.**
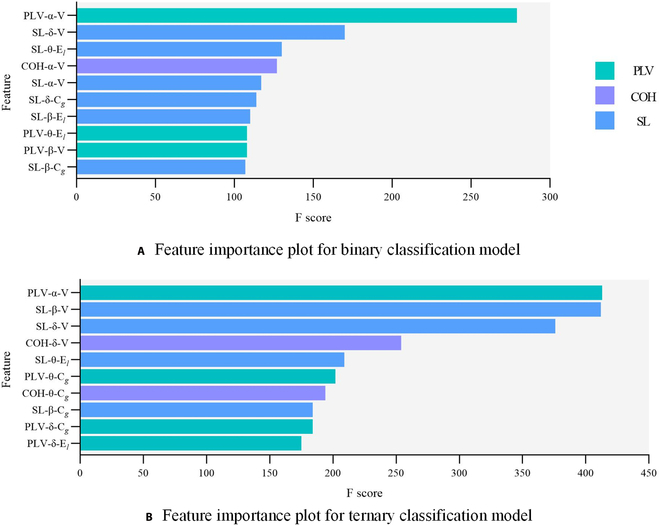
Feature importance ranking in XGBoost model: (A) for binary classification, (B) for ternary classification. V, connectivity strength; C_g_, clustering coefficients; E_l_, local efficiency.

From the perspective of classification performance, the features’ contributions of different networks could be extracted and might provide further insight on the feature engineering of distracted driving state recognition. Figure 9 depicts the feature importance ranking for classifying different driving states using XGBoost model, revealing which combinations of features derived from network analysis properly contribute to the augmented recognition. Irrespective of binary classification (distinguishing between normal and distracted driving states) or ternary classification (classifying normal, cognitive distraction, and visual distraction states), the connectivity strength of PLV network in α band attains the highest scores, and the connectivity strength of SL network in δ band and local efficiency of SL network in θ band demonstrate consistent high importance.

Additionally, when examining the foremost 5 features of the 2 classification models, it is observed that the connectivity strength of SL network and COH network in α band exhibits notable significance in the binary classification task. Comparatively, the connectivity strength of SL network in β band and COH network in δ band manifest heightened importance in the ternary classification task. Considering the 3 network features collectively, the SL network feature exhibits the highest prominence, constituting 54.60% of the score in the binary classification model and 45.37% in the ternary classification model as indicated in the provided importance scores. This further underscores the superior discriminatory capability of SL network features in identifying driving states compared to other networks. Moreover, a noteworthy observation is the significant contribution of local efficiency (E*_l_*) and clustering coefficient (C*_g_*) features to the augmented recognition of driving states, suggesting their susceptibility to fluctuations in cognitive and visual resource allocation.

In light of the current model performance and findings, there exist promising research directions for future enhancements and advancements. First, our study exclusively focuses on the distracted driving states during the car-following process and does not address other scenarios involving lane changes and turns. Given the limited duration of simulator experiments and the complex stimuli of distracted tasks in real-world driving, it is conceivable that there exist latent factors that may exert disparate effects on the brain network features. In future research, it is essential to consider more nuanced distracted factors and situations from the real life. Second, although 3 connectivity indices and 4 topological features were employed to portray the functional brain networks, it is also valuable to explore the effect of other promising synchronization indicators and SPN features in future research. Moreover, investigating the structural information of predefined brain regions of interest (ROIs) through diffusion tensor imaging (DTI) could promote structural–functional fusion representation of brain network [58,59]. Third, EEG signals usually present low signal-to-noise ratio. Facing this inherent challenge, effective artifact removal methods [60] or combining EEG with other complementary recording modalities such as fMRI can be employed to enhance feasibility and practicality of the proposed method. Furthermore, by integrating relevant prior knowledge with the collected data may significantly improve the model’s generalizability [61]. Fourth, our work mainly focused on the driver distraction during human driving. The automation of vehicles has afforded drivers a greater scope of distraction while simultaneously introducing potential risks associated with the possible takeovers [62]. An essential focus of future research involves analyzing the driver distraction state during autonomous driving and exploring the potential for human–machine integration in multitask scenarios based on brain activity [63].

### Conclusion

Electroencephalography (EEG) can naturally measure physiological responses under distracted driving conditions. In this study, we collected the EEG signals of drivers to construct varied brain functional networks using SL, PLV, and coherence (COH) connectivity metrics. The networks’ connection strength and topological features including clustering coefficients, characteristic path lengths, global efficiency, and local efficiency were extracted to classify the driving states. The research intuitively reveals the network topological differences among normal, cognitively distracted, and visually distracted driving states and attempts to optimize the model performance of state recognition. The results indicate the following: (a) The configuration of the functional brain network is associated with the driver’s distracted state, suggesting increased activity within the frontal lobe under distraction, especially at theta and beta bands. (b) Among the 3 network types, SL network features demonstrate superior recognition capability for distracted driving states compared to other networks. (c) The combined features of the 3 networks exhibit the best classification performance on binary classification of distracted driving states, as well as on recognizing normal, cognitively, and visually distracted driving states, with XGBoost demonstrating the highest accuracy of 95.1% and 88.2%, respectively. The primary contribution of this study lies in the establishment of a driving state augmented recognition framework based on brain network features, not only for binary classification of distraction but also for more delicate division of cognitive and visual distraction. Such electrophysiological analysis of brain network will provide a foundation for the advancement of driver assistance systems with distraction control strategies and development of brain-controlled systems, in both conventional human driving scenarios and autonomous driving contexts.

## Data Availability

The datasets used and/or analyzed during the current study are available from the corresponding author on reasonable request.
